# Vacancy-engineered nodal-line semimetals

**DOI:** 10.1038/s41598-022-18519-8

**Published:** 2022-09-02

**Authors:** Fujun Liu, Fanyao Qu, Igor Žutić, Mariana Malard

**Affiliations:** 1 Nanophotonics and Biophotonics Key Laboratory of Jilin Province, School of Physics, Changchun University of Science and Technology, Changchun, 130022 People’s Republic of China; 2 Instituto de Física, Universidade de Brasília, Brasília, DF 70904-910 Brazil; 3 The State University of New York, University at Buffalo, Buffalo, NY 14260 USA; 4 Faculdade UnB Planaltina, Universidade de Brasília, Brasília, DF 70904-910 Brazil

**Keywords:** Topological matter, Electronic structure

## Abstract

Symmetry-enforced nodal-line semimetals are immune to perturbations that preserve the underlying symmetries. This intrinsic robustness enables investigations of fundamental phenomena and applications utilizing diverse materials design techniques. The drawback of symmetry-enforced nodal-line semimetals is that the crossings of energy bands are constrained to symmetry-invariant momenta in the Brillouin zone. On the other end are accidental nodal-line semimetals whose band crossings, not being enforced by symmetry, are easily destroyed by perturbations. Some accidental nodal-line semimetals have, however, the advantage that their band crossings can occur in generic locations in the Brillouin zone, and thus can be repositioned to tailor material properties. We show that lattice engineering with periodic distributions of vacancies yields a hybrid type of nodal-line semimetals which possess symmetry-enforced nodal lines and accidental nodal lines, with the latter endowed with an enhanced robustness to perturbations. Both types of nodal lines are explained by a symmetry analysis of an effective model which captures the relevant characteristics of the proposed materials, and are verified by first-principles calculations of vacancy-engineered borophene polymorphs. Our findings offer an alternative path to relying on complicated compounds to design robust nodal-line semimetals; one can instead remove atoms from a common monoatomic material.

## Introduction

The study of degeneracies between energy bands in the spectrum of a system dates back to the early days of quantum mechanics^1^. Since then, band degeneracies are featured in various physical phenomena, from signaling quantum phase transitions^2^ to being the spectral signature of a semimetal^3,4^. According to the non-crossing rule by von Neumann and Wigner^1^, energy bands generally avoid each other, but symmetries entail the possibility of band crossings. A band crossing is *symmetry-enforced* if its existence is guaranteed by the underlying symmetry(ies) alone, and *accidental* if the band crossing requires also tuning parameters of the material (e.g. the hopping energy, or any other microscopic parameter of the material). A special case occurs in three dimensions where the availability of sufficiently many tunable parameters (three momentum coordinates and one material parameter) leads to accidental band crossings even in the absence of symmetries^3^. Symmetry-enforced band crossings arise from nonsymmorphic symmetries (point group transformations followed by a non-primitive translation), as first realized by Michel and Zak in 1999^5^, and later applied to the research on topological semimetals (TSMs)^6–8^.

The nontrivial topology of band crossings in TSMs underpins a variety of phenomena, e.g. Fermi arcs^9^ and chiral anomaly^10^, and promise diverse technological applications, notably in topological quantum computing^4^ and in quantum-device designs based on proximity effects^11^. TSMs with accidental band crossings are topologically protected only locally, being eventually destroyed by perturbations. In contrast, TSMs with symmetry-enforced band crossings are endowed with global topological protection, i.e. they are immune to symmetry-preserving perturbations^6,7^.

Here we focus our attention on nodal-line semimetals (NLSMs), the TSMs with band crossings occurring along lines in the Brillouin zone (BZ). Two-dimensional (2D) structures, e.g. hexagonal lattices^12^ and honeycomb-Kagome lattices^13^, and PbFCl-type structures^14^, have been predicted to realize NLSMs. However, the frailty to perturbations, particularly to spin-orbit coupling (SOC), of the resulting accidental nodal lines (NLs) hinders possible applications and use of proximity effects^11^. Symmetry-enforced NLSMs are the natural candidates to overcome this challenge. Predicted realizations comprise 3D materials from hexagonal groups $P\bar{6}2c$, $P6_{1}22$, and $P6_{3}/m$^15^, and group *P*4/*nmm*^16^. A recent review gathers the most recent developments in the quest for 3D NLSMs^17^.

As an alternative to naturally nonsymmorphic 3D crystals, we propose to engineer 2D symmetry-enforced NLSMs by turning symmorphic monoatomic sheets into nonsymmorphic ones through removing atoms. Quite surprisingly, besides symmetry-enforced NLs, vacancy-engineered materials exhibit also accidental NLs which survive under very strong SOC, with strength even exceeding the experimentally attainable values. These additional NLs were first noted in our previous report^18^ where their robustness was conjectured to be symmetry enforced since accidental NLs had so far been known to be easily destroyed by perturbations. However, these NLs can be moved inside the Brillouin zone (BZ) by varying the parameters of the material, contradicting a known feature of symmetry-enforced NLs, namely, that they are pinned at high-symmetry momenta in the BZ. Our movable, and yet unusually robust, NLs do not fit in either. one of the two known categories of NLs: robust and fixed symmetry-enforced NLs and fragile and movable accidental NLs. We thus uncover that the usual approach to distinguish NLs by their response to perturbations is inadequate when such band crossings appear simultaneously in the spectrum and have a common origin.

In this work, we develop a complete theoretical description of a 2D vacancy-engineered nonsymmorphic lattice which resolves the puzzle by demonstrating that, despite their unusual robustness, the NLs which are movable in the interior of the BZ are accidental, and that symmetry-enforced NLs also exist, but are pinned at an edge of the BZ. While the accidental NLs are not directly wielded by the nonsymmorphic symmetry per se, they are a direct consequence of the proposed mechanism of attaining nonsymmorphicity out of vacancies. The fact that these accidental NLs can move and change shape inside the BZ might enable manipulation of momentum-dependent scattering processes and, hence, of various responses of the material.

Our analysis based on a 2D four-band effective model adds to the known proofs of band-degeneracy enforcement for a 1D two-band model^6,7^ and for a 2D two-band model^8^. It is well-known that a glide-plane symmetry enforces band degeneracies at the glide-invariant boundary of the BZ. But whether this band degeneracy is a nodal point or a NL is not guaranteed by the glide-plane symmetry. In Refs.^6–8^ the nonsymmorphic symmetry yields nodal points located at BZ corners. In contrast, our formalism is specifically focused on symmetry-enforced NLs along an edge of a 2D BZ. This fundamental difference is connected to the roles played by inversion and time-reversal symmetries in the effective models, which depend on dimensionality and number of bands. The NLs uncovered here are to be contrasted also with those predicted for 3D nonsymmorphic materials^15,16^. Symmetry-enforced NLs in 2D thus constitute a new case of band-degeneracy enforcement. The proposed mechanism of engineering nonsymmorphic 2D lattices from periodic configurations of vacancies offers an alternative path to fundamental investigations and materials design of NLSMs.

## Results

### Material realizations

**Figure 1:**
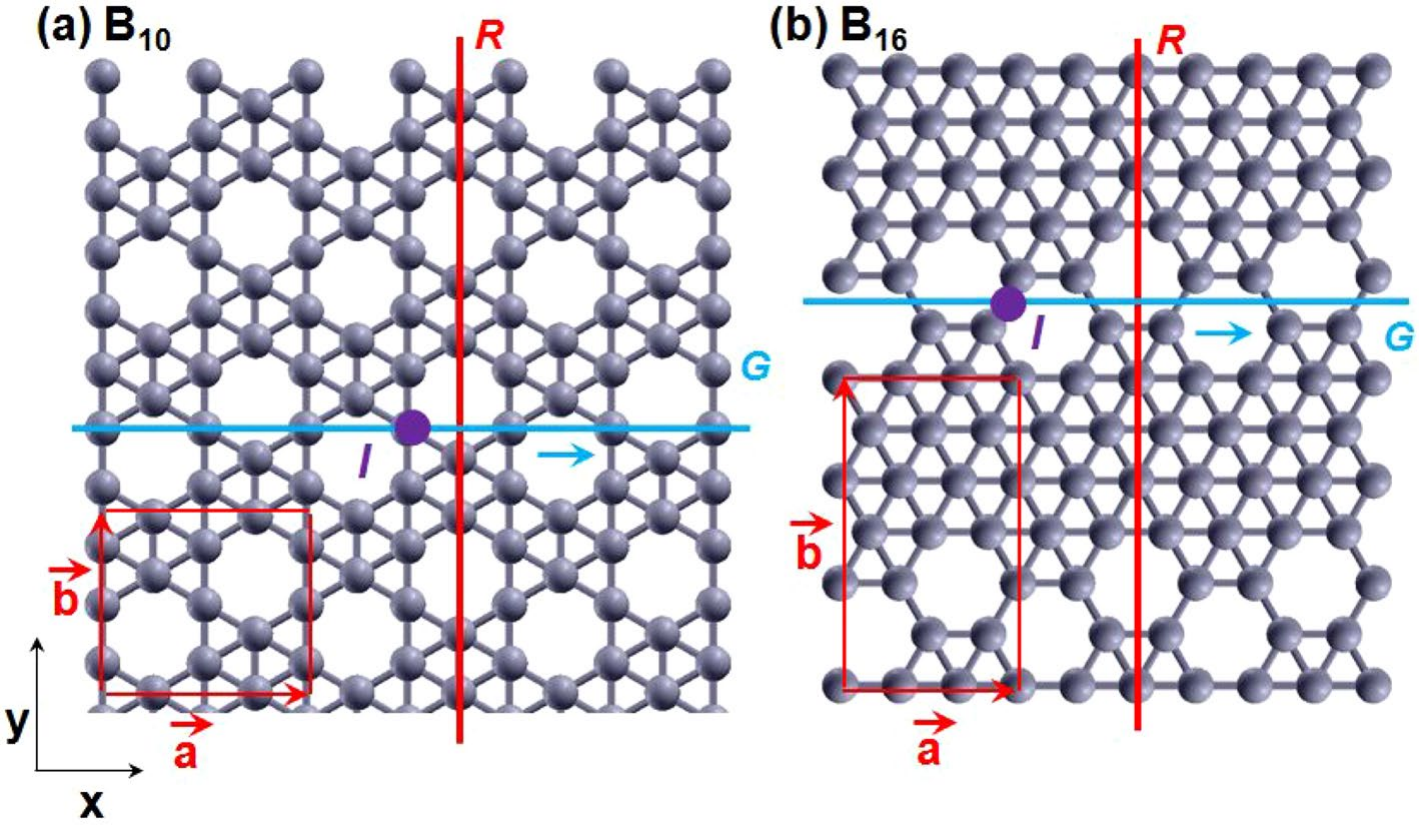
Lattice structure of borophene B$_{10}$ (**a**) and B$_{16}$ (**b**) with 10 and 16 atoms, respectively, in the unit cell defined by primitive vectors $\vec {a}$ and $\vec {b}$. B$_{10}$ [B$_{16}$] is obtained from pristine borophene by removing boron atoms from the center of hexagons which share one corner [side] along the *x*-direction. In both (**a,b**), the hollow hexagons form a string along the *x*-direction, with a zigzag profile in the *y*-direction. This basic feature yields a nonsymmorphic glide-plane symmetry *G* (composed of a reflection plane running along the *x*-direction and a non-primitive translation by $\vec {a}/2$), a symmorphic reflection-plane symmetry *R* perpendicular to *G*, and a symmorphic inversion-point symmetry *I*.

As a concrete example, we illustrate our idea in borophene, a 2D lattice of boron atoms. The character of boron bonding—with short covalent radius and possibility of $sp^{2}$ hybridization—favors the formation of a plethora of low-dimensional allotropes, including sheets with different crystalline motifs^19–33^. In the first reported realizations, different borophene sheets were grown on a Ag (111) substrate through electron beam evaporation^23^ and molecular beam epitaxy^24^. Subsequent developments in the growth and stabilization of borophenes were compiled in a recent review ^32^. It reports a wealth of experimentally realized boron 2D lattices with periodic distributions of vacancies, and the agreement between first-principles predictions and experimental realizations for various vacancy concentrations. We can thus expect different robust NLSM-designs using borophene as a base material. Here we propose and investigate the two stable borophenes shown on Figure 1a,b, denoted as B$_{10}$ and B$_{16}$, where the subscripts refer to the number of atoms in the unit cell. B$_{10}$ and B$_{16}$, which belong to the *pmg* nonsymmorphic wallpaper group^34^, feature periodic patterns of vacancies which are similar to the ones experimentally obtained in Ref.^24^.

Pristine borophene without any vacancies is a symmorphic material; it possess two perpendicular reflection planes which entail the appearance of accidental Dirac cones in the spectrum, akin to those in graphene, silicene and germanene. Introducing vacancies at proper concentrations and configurations in pristine borophene yields that one of the reflection planes is replaced by a nonsymmorphic glide plane (c.f. Fig. 1a,b), and the Dirac cones give place to symmetry-enforced NLs. On top of that, the spectrum acquires also accidental NLs which are robust to strong Rashba SOC.

### Symmetry-enforced band degeneracies of a nonsymmorphic two-dimensional lattice

**Figure 2:**
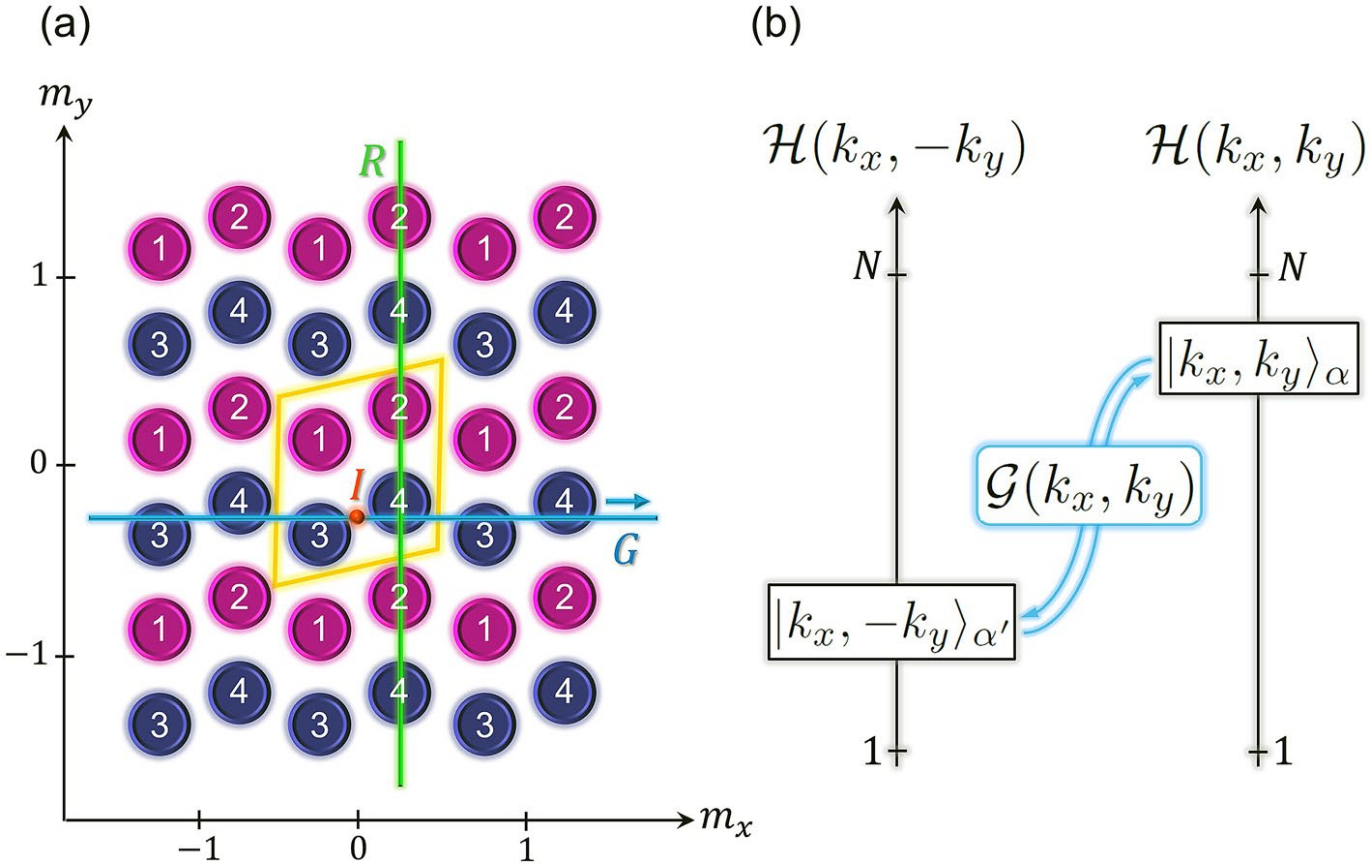
(**a**) A minimal nonsymmorphic two-dimensional lattice, with the unit cell delineated by the yellow lines. The location of the unit cell is defined by $(m_{x},m_{y})$. The disks numbered from 1 to 4 represent two types of structures within the unit cell. In real borophenes B$_{10}$ and B$_{16}$ shown in Fig. 1a,b, these structures are the hollow and filled pieces that make up the unit cell of those materials. The minimal lattice has the following spatial symmetries: a nonsymmorphic glide plane, *G*, composed of a reflection about the $m_{x}$-direction, followed by a nonprimitive translation by half of the length of the unit cell along the $m_{x}$-direction, a symmorphic reflection plane, *R*, about the $m_{y}$-direction, and a symmorphic inversion point, *I*, which takes a point $\vec {r}$ on the lattice to $-\vec {r}$. (**b**) The glide-plane symmetry $\mathscr{G}(k_{x},k_{y})$ transforms the $|k_{x},k_{y}\rangle _{\alpha }$ eigenstate of the Bloch Hamiltonian $\mathscr{H}(k_{x},k_{y})$ into the $|k_{x},-k_{y}\rangle _{\alpha '}$ eigenstate of $\mathscr{H}(k_{x},-k_{y})$, and vice-versa. *N* is the number of bands ($N=4$ for the lattice depicted in (**a**)).

In this section we carry out a symmetry analysis of a minimal lattice which captures the symmetries of B$_{10}$ and B$_{16}$ shown in Fig. 1a,b. Figure 2a depicts a 2D lattice whose unit cell has four internal degrees of freedom, represented by the magenta and blue disks which are shifted along the $m_{y}$-direction. This shift mimics the profile of the filled and hollowed stripes in B$_{10}$ and B$_{16}$. The minimal lattice is invariant under a nonsymmorphic glide plane *G*, a symmorphic reflection plane *R*, and a symmorphic inversion point *I*. We now analyze how these symmetries constrain the band structure of the $4\times 4$ Bloch Hamiltonian, $\mathscr{H}(k_{x},k_{y})$, of the lattice depicted in Fig. 2a. This analysis is sample-independent; it relies only on the symmetries of the minimal lattice shown in Fig. 2a.

The invariance of the lattice with respect to the glide-plane transformation *G* is manifest in the relation

1 $$\begin{aligned} \mathscr{G}(k_{x},k_{y})\mathscr{H}(k_{x},-k_{y})\mathscr{G}^{-1}(k_{x},k_{y})=\mathscr{H}(k_{x},k_{y}), \end{aligned}$$

where $\mathscr{G}(k_{x},k_{y})$ is the $4 \times 4$ matrix representation of *G* in the basis constructed by the eigenstates of $\mathscr{H}(k_{x},k_{y})$. The $k_{x}$-dependance of $\mathscr{G}$ stems from the fractional translation along the $m_{x}$-direction, while the $k_{y}$-dependance originates from the shift of the glide plane from the center of the unit cell along the $m_{y}$-direction (c.f. Fig. 2a). So *G* is an unusual symmetry which is both nonsymmorphic (along $m_{x}$) and off-centered^35,36^ (along $m_{y}$). Such a glide plane differs from the one used to prove band-degeneracy enforcement in a 1D two-band model, the latter being a $2 \times 2$ matrix which depends on only one momentum coordinate^6,7^.

**Figure 3:**
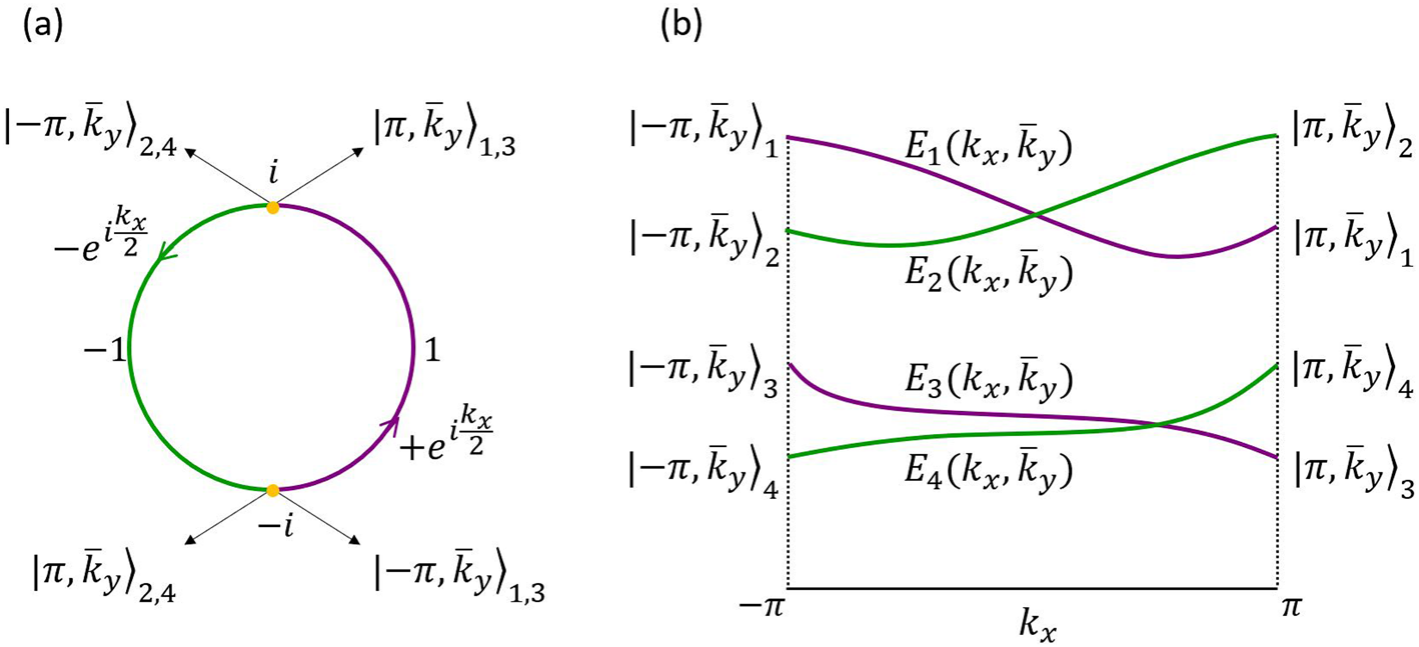
(**a**) Doubly-degenerate eigenvalues $\pm e^{i\frac{k_{x}}{2}}$ of the glide-plane matrix winding around the half-unit circles on the complex plane, one pair of eigenvalues from $-i$ to *i* through 1, and the other pair from *i* to $-i$ through − 1. The eigenstates $|\pm \pi ,\bar{k}_{y}\rangle _{1,3}$ and $|\pm \pi ,\bar{k}_{y}\rangle _{2,4}$ associated to the extreme points of the eigenvalue-trajectories are indicated. (**b**) The behavior of the eigenvalues of the glide-plane matrix implies that the eigenvalues of the Bloch Hamiltonian must cross pairwise at some value of $k_{x}$.

We show (c.f. Sect. 1 of the Supporting Information) that $\mathscr{G}(k_{x},k_{y})|k_{x},-k_{y}\rangle _{\alpha '}\rightarrow |k_{x},k_{y}\rangle _{\alpha }$, where $|k_{x},k_{y}\rangle _{\alpha }$ ($\alpha =1,2,3,4$) is a Bloch eigenstate of $\mathscr{H}(k_{x},k_{y})$. This transformation between the negative-$k_{y}$ and positive-$k_{y}$ Bloch eigenspaces is illustrated in Fig. 2b. On the lines $k_{y}=\bar{k}_{y}=0,\pm \pi$, $\mathscr{H}(k_{x},-\bar{k}_{y})=\mathscr{H}(k_{x},\bar{k}_{y})$. Equation (1) thus yields $[\mathscr{G}(k_{x},\bar{k}_{y}),\mathscr{H}(k_{x},\bar{k}_{y})]=0$ and, hence, $|k_{x},\bar{k}_{y}\rangle _{\alpha }$ are also eigenstates of $\mathscr{G}(k_{x},\bar{k}_{y})$. By constructing the matrix $\mathscr{G}(k_{x},k_{y})$ (c.f. Sect. 1 of the Supporting Information), we obtain the twofold degenerate eigenvalues of $\mathscr{G}(k_{x},\bar{k}_{y})$: $\xi _{1,3}=\,\text {exp}(ik_{x}/2)$, and $\xi _{2,4}=-\,\text {exp}(ik_{x}/2)$. As $k_{x}$ swipes the BZ from $-\pi$ to $\pi$, the $(+)$-eigenvalues wind around the half-unit circle on the complex plane from $-i$ to *i* through 1, while the $(-)$-eigenvalues wind from *i* to $-i$ through − 1, as illustrated on Fig. 3a. As a result, the eigenstates $|-\pi ,\bar{k}_{y}\rangle _{1,3}$ and $|\pi ,\bar{k}_{y}\rangle _{2,4}$ have the same $\mathscr{G}$-eigenvalue, $-i$, and the eigenstates $|\pi ,\bar{k}_{y}\rangle _{1,3}$ and $|-\pi ,\bar{k}_{y}\rangle _{2,4}$ have the same $\mathscr{G}$-eigenvalue, *i*. It follows that the associated pairs of $\mathscr{H}$-eigenvalues must cross at least once along the $k_{x}$-axis^6^ (when $k_{y}=\bar{k}_{y}$), as shown in Fig. 3b. From this analysis we conclude that the glide-plane symmetry enforces the appearance of two nodal points at $k_{y}=0$ and two nodal points at $k_{y}=\pm \pi$. These nodal points, which were not unveiled in Ref.^18^, are the precursors of symmetry-enforced NLs.

To further clarify the origin of the nodal points depicted in Fig. 3b, we analyze the simplest tight-binding model for the lattice shown in Fig. 2a in which only hopping between nearest-neighbor sites and on-site energies are considered. The entries of the corresponding Bloch Hamiltonian $\mathscr{H}(k_{x},k_{y})$ read

2 $$\begin{aligned} \begin{aligned} \varepsilon _{n,m}(k_{x},k_{y}) =& t_{n,m}+t_{m,n}^{*}+u_{n,m}e^{ik_{x}}+u_{m,n}^{*}e^{-ik_{x}}+v_{n,m}e^{ik_{y}}\\&+v_{m,n}^{*}e^{-ik_{y}}+w_{n,m}e^{i(k_{x}+k_{y})}+w_{m,n}^{*}e^{-i(k_{x}+k_{y})}, \end{aligned} \end{aligned}$$

where $n,m=1,...,4$, and $t_{n,m}$, $u_{n,m}$, $v_{n,m}$, and $w_{n,m}$ denote the hopping energy from site *m* to *n* within the same unit cell, between neighboring unit cells along the *x*-direction, *y*-direction, and diagonal direction, respectively, and with $t_{n,n}=\mu _{n}$ the on-site energy. In Eq. (2), only $u_{2,1}=t_{2,1}$, $u_{4,1}=t_{4,1}$, $u_{4,3}=t_{4,3}$ along *x*, $v_{1,3}=t_{1,3}$, $v_{2,3}=t_{2,3}$, $v_{2,4}=t_{2,4}$ along *y*, and $w_{2,3}=t_{2,3}$ along the diagonal are non-vanishing neighboring hopping (c.f. Fig. 2a).

Imposing Eq. (1), with $\mathscr{G}(k_{x},k_{y})$ given by Eq. (S5), leads to Eqs. (S8)–(S11) (c.f. Sect. 1 of the Supporting Information) constraining the entries $\varepsilon _{n,m}(k_{x},k_{y})$ of $\mathscr{H}(k_{x},k_{y})$. A similar argument to the one used for the off-diagonal entry of a 1D two-band model^6^ can be applied here to show that Eq. (S9) implies that $\varepsilon _{n,n+1}(k_{x},\bar{k}_{y})$, $n=1,3$, must vanish at some value of $k_{x}$. In a 1D two-band model with only one off-diagonal entry, its vanishing is sufficient to guarantee a band crossing. In a four-band model, on the other hand, the vanishing of only two of its off-diagonal entries is not a sufficient condition. For a 1D multi-band model, band crossings occur provided that the model has, on top of the nonsymmorphic symmetry, also chiral symmetry^6^. Chiral symmetry means that the Bloch Hamiltonian admits an off-diagonal block form which, in turn, means that half of its entries are identical to zero. This is clearly not the case of our 2D four-band $\mathscr{H}(k_{x},k_{y})$ with entries given by Eq. (2), which allows for hopping between any two intracell sites and on-site energies. This is particularly relevant for making a connection with a real 2D material in which hopping occurs in all directions. Therefore, the (nonsymmorphic + chiral)-symmetries argument designed for the 1D multi-band case is not applicable here.

**Figure 4:**
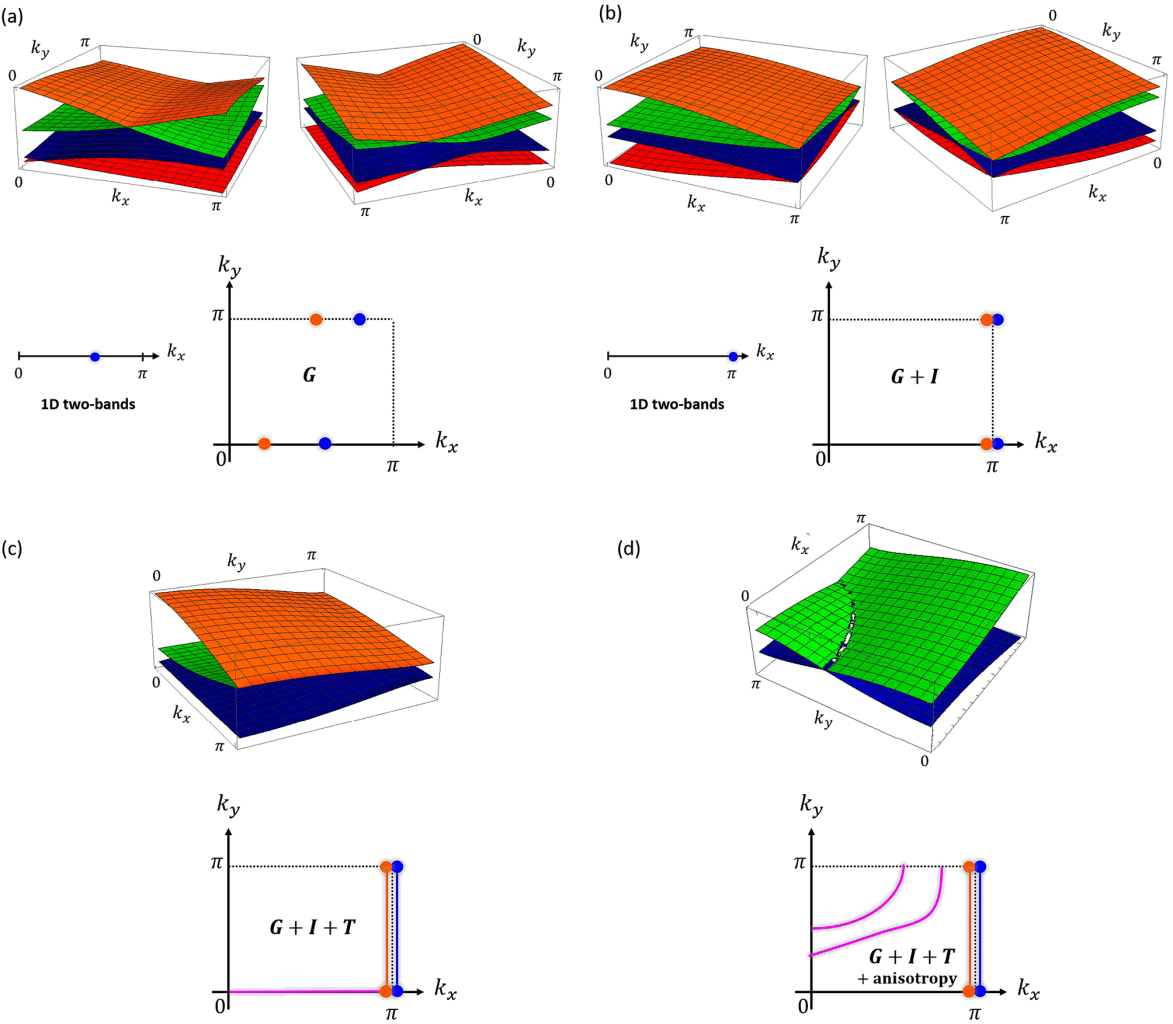
(**a–d**) Upper panels: Energy bands of the effective model with glide-plane (G) symmetry, with glide-plane (G) + inversion-point (I) symmetries, with glide-plane (G) + inversion-point (I) + time-reversal (T) symmetries, and with glide-plane (G) + inversion-point (I) + time-reversal (T) symmetries + anisotropy, respectively. (**a–d**) Lower panels: Projection on the $k_{x}-k_{y}$ plane of the nodal points or nodal lines shown on the corresponding upper panels. The lower panels of (**a,b**) contain also the 1D two-band result for comparison. The lower panel of (**d**) illustrates two accidental nodal lines inside the Brillouin zone, corresponding to different anisotropic cases.

Instead, we must impose all Eqs. (S8)–(S11) on the entries given by Eq. (2). This yields the entries constrained by the glide-plane symmetry given by Eqs. (S12)–(S17) (c.f. Sect. 1 of the Supporting Information). The upper panel of Fig. 4a shows two views of the band structure of the glide-plane invariant effective model given by Eqs. (S12)–(S17) on the positive quadrant of the $k_{x}-k_{y}$ plane, for $\mu _{1}=\mu _{3}=0$ and $t_{2,1}=t_{4,3}=t_{1,3}=t_{3,1}=t_{1,4}=t_{4,1}=\text {exp}(i0.3\pi )$ (with $\mu$’s and *t*’s given in arbitrary units). The bands touch pairwise at some value of $k_{x}$, provided $k_{y}=\bar{k}_{y}=0,\pi$. The lower panels of Fig. 4a depicts the projection of the nodal points on the $k_{x}-k_{y}$ plane, with the 1D two-band model result^6^ shown on the left for comparison. Including higher-order hopping in Eq. (2) will move the glide-plane enforced nodal points along $k_{x}$, but will not gap them out since higher-order hopping preserve the glide-plane symmetry. We thus see that exact diagonalization of the effective model confirms the previous prediction derived from the general relations obeyed by the eigenvalues of $\mathscr{H}(k_{x},k_{y})$ and $\mathscr{G}(k_{x},k_{y})$, namely, that the glide-plane symmetry enforces two pairs of nodal points, one pair at $k_{y}=0$ and another at $k_{y}=\pm \pi$. Having the energy spectrum and the corresponding single-particle states wielded by exact diagonalization, one can form the density matrix and compute thermodynamic averages or, using linear response theory, extract transport properties. For the present purpose of demonstrating the existence of NLs, next we show how the remaining symmetries turn *G*-enforced two pairs of nodal points into two NLs pinned at the $k_{x}=\pi$ edge of the BZ.

### Effect of inversion and time-reversal symmetries

The invariance relation representing the symmetry of the lattice with respect to the inversion-point transformation *I* is

3 $$\begin{aligned} \mathscr{I}(k_{y})\mathscr{H}(-k_{x},-k_{y})\mathscr{I}^{-1}(k_{y})=\mathscr{H}(k_{x},k_{y}), \end{aligned}$$

where $\mathscr{I}(k_{y})$ is the $4 \times 4$ matrix representation of *I* in the basis constructed by the eigenstates of $\mathscr{H}(k_{x},k_{y})$. Similar to what happens with $\mathscr{G}$, the $k_{y}$-dependance of $\mathscr{I}$ is a consequence of the shift of *I* from the center of the unit cell along the $m_{y}$-direction (c.f. Fig. 2a).

Combining the constraints imposed by Eq. (3) with those from Eq. (1), we obtain Eqs. (S27)–(S31) (c.f. Sect. 2 of the Supporting Information) which give the off-diagonal entries of the glide-plane and inversion-point invariant effective model. The resulting band structure and projection of nodal points on the $k_{x}-k_{y}$ plane are shown on the upper and lower panels of Fig. 4b for $\mu _{1}=\mu _{3}=0$, $t_{2,1}=t_{4,3}=1$ (*I*-symmetry implies that these hopping parameters must be real; c.f. Sect. 2 of the Supporting Information), and $t_{1,3}=t_{3,1}=t_{1,4}=\text {exp}(i0.3\pi )$. Figure 4b conveys that the effect of the inversion-point symmetry is to pin at $k_{x}=\pi$ the four nodal points of the glide-plane invariant effective model. This behavior should be distinguished from that in Ref.^18^, where the inversion-point combines with a last local constraint on the Hamiltonian to generate NLs at $k_{x}=\pi$. Within the present framework, the influence of the inversion-point symmetry alone, i.e. with no additional constraints, is simply to push the nodal points enforced by the glide-plane symmetry to $k_{x}=\pi$. The NLs emerge only when invoking time-reversal symmetry, as we shall see next. For completeness, the known result from the 1D two-band model is shown on the left side of the lower panel of Fig. 4b. It can be shown that *G* and *I* imply *R* (c.f. Sect. 3 of the Supporting Information). Therefore, it is sufficient to analyze the constraints imposed by *G* and *I* on the band structure.

Turning to the time-reversal transformation *T*, not considered in Ref.^18^ (where the focus was on breaking time-reversal symmetry by magnetic proximity effect), the invariance relation is given by

4 $$\begin{aligned} \mathscr{T}\mathscr{H}^{*}(-k_{x},-k_{y})\mathscr{T}^{-1}=\mathscr{H}(k_{x},k_{y}), \end{aligned}$$

where $\mathscr{T}=1\!\!1_{4 \times 4}$ since $\mathscr{H}(k_{x},k_{y})$ is spinless.

Imposing Eq. (4) implies that the entries of $\mathscr{H}(k_{x},k_{y})$ satisfy $\varepsilon ^{*}_{n,m}$(-$k_{x}$, -$k_{y}$)$= \varepsilon _{n,m}(k_{x},k_{y})$ which, given Eq. (2), yields real hopping parameters. As can be seen in the upper and lower panels of Fig. 4c, in which $\mu _{1}=\mu _{3}=0$, $t_{2,1}=t_{4,3}=t_{1,3}=t_{3,1}=t_{1,4}=1$, the effect of time-reversal symmetry on the band structure of the glide-plane and inversion-point invariant effective model is twofold: it connects the symmetry-enforced nodal points of the same pair of bands, thus forming two NLs at $k_{x}=\pi$, and it also induces an additional NL at $k_{y}=0$. While the former NLs are symmetry-enforced, the later is accidental, being gapped, for instance, by an anisotropy of the hopping parameters, as we shall see next.

### Effect of anisotropy

Here we discuss another feature of the band structure which is special to our scheme of engineering a nonsymmorphic symmetry out of vacancies: The appearance of unusually robust accidental NLs in the interior of the BZ. Accidental band crossings are usually very fragile to perturbations or change in material parameters. The appearance of NLs inside the BZ which survived extremely strong SOC and magnetic exchange field (of strengths nearing or exceeding current experimental bounds) was initially conjectured, in Ref.^18^, to come from symmetry enforcement. The framework developed here conclusively resolves this puzzle and identifies the origin of such unusually robust, yet accidental, NLs.

In a pristine monoatomic lattice in which the distance between neighboring atoms is the same in all directions (such as borophene), the hopping amplitudes are direction-independent. In such an isotropic environment (and disregarding non-structural degrees of freedom such as orbitals and spin), the crossings between energy bands are the ones associated only to the symmetries, as we have seen in the previous sections. Defects (including vacancies) break the isotropy of the hopping amplitudes, with the result that now bands can cross also in generic places of the BZ. For the particular band structure shown in Fig. 4c, the anisotropy gaps out the accidental NL at $k_{y}=0$, but creates another accidental NL between the middle bands in the interior of the BZ, as shown on the upper panel of Fig. 4d where $\mu _{1}=\mu _{3}=0$, $t_{2\,1}=t_{4\,3}=1$, and $t_{1\,3}=t_{3\,1}=t_{1\,4}=100$. Smoothly changing the anisotropy between the hopping amplitudes makes the NL move and change shape through the BZ, as illustrated on the lower panel of Fig. 4d. Eventually, the NL starts to fade and finally disappears when the parameters are taken out of a certain anisotropic regime. Unlike accidental NLs of nonstructural origin, which are easily gapped by perturbations, anisotropy-induced accidental NLs should have an enhanced robustness owing to their structural origin. This feature can be traced to the fact that perturbations do not restore isotropy (sometimes they might actually enhance the anisotropy). Finally, while anisotropy in the Hamiltonian parameters induces accidental NLs inside the BZ, this is not a sufficient condition. The appearance of such NLs is preconditioned by the presence of time reversal symmetry. We will further elaborate on this point in the next section where the shapes of the accidental NLs of vacancy-engineered borophenes signal their connection to time-reversal symmetry.

We conclude that a nonsymmorphic 2D material created by vacancy-engineering possesses symmetry-enforced NLs at one edge of the BZ originated from a glide-plane symmetry (combined with inversion-point and time-reversal symmetries), and also accidental NLs with enhanced robustness in the interior of the BZ from the vacancy-induced anisotropy and time-reversal symmetry.

### Density functional theory calculation

**Figure 5:**
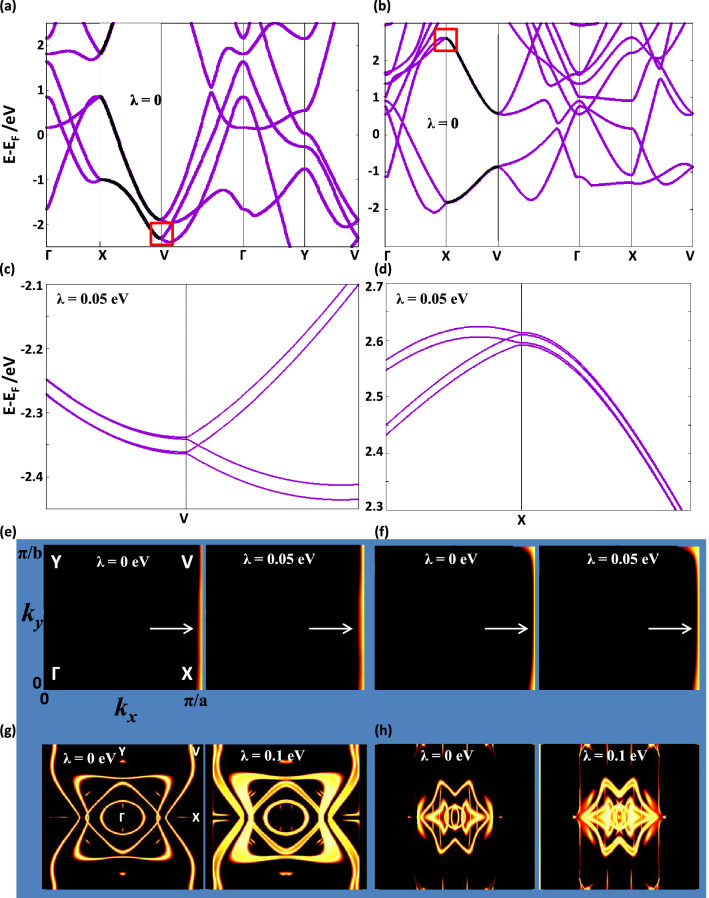
(**a,b**) Band structure of B$_{10}$ [B$_{16}$] without Rashba spin-orbit coupling (SOC) ($\lambda =0$ eV) along the $\Gamma$-X-V-$\Gamma$-Y-V path in the Brillouin zone, with the position of points $\Gamma$, X, V, and Y given in the left panel of (**e**). The symmetry-enforced nodal lines along the X-V direction are highlighted in black. (**c,d**) Amplified image of the bands inside the red rectangles in (**a,b**), but in the presence of Rashba SOC ($\lambda =0.05$ eV). Each fourfold degenerate symmetry-enforced nodal line splits into a pair of spin-split twofold degenerate nodal lines. (**e,f**) Contour plot of the symmetry-enforced nodal lines shown in (**a,b**) without Rashba SOC in the left panel ($\lambda =0$ eV) and in the presence of Rashba SOC in the right panel ($\lambda =0.05$ eV). The nodal lines, which are pinned at the edge $k_{x}=\pi /a$ of the Brillouin zone, are indicated by arrows. (**g,h**) Contour plot of all accidental nodal lines which exist within an energy window of 2.0 eV about the Fermi energy in the band structure shown in (**a,b**), without Rashba SOC in the left panel ($\lambda =0$ eV) and in the presence of Rashba SOC in the right panel ($\lambda =0.1$ eV).

Figure 5a,b show the band structure of B$_{10}$ and B$_{16}$, respectively, both without Rashba SOC ($\lambda =0$). The bands are given along the $\Gamma$-X-V-$\Gamma$-Y-V path in the BZ (points $\Gamma$, X, V, and Y are shown on the left panel of Fig. 5e). Figure 5a,b indicate that bands stick together pairwise, forming NLs, along the X-V direction (corresponding to $k_{x}=\pi /a$). Figure 5c,d show the amplified image of the bands inside the red rectangles in Fig. 5a,b, respectively, but in the presence of Rashba SOC ($\lambda =0.05$ eV). Figure 5e,f, left panels, are the contour plots in the $k_{x}-k_{y}$ plane of the NLs shown in Fig. 5a,b, respectively. In these contour plots, the orange line running along X-V represents the vanishing of the energy difference between the sticking bands. Figure 5g,h, left panels, are the contour plots of the NLs which exist in the interior of the BZ of the band structures shown in Fig. 5a,b, respectively, within an energy window of 2.0 eV about the Fermi energy. Figure 5e–h, right panels, show the contour plots of the NLs in the corresponding left panes but with Rashba SOC of strength $\lambda =0.05$ eV in Fig. 5e–f and $\lambda =0.1$ eV in Fig. 5g,h.

Rashba SOC preserves the crystalline symmetries, as well as time-reversal symmetry. The NLs featured in the dispersions, shown in Fig. 5a–d, which are projected on the $k_{x}-k_{y}$ plane in Fig. 5e,f, correspond to those of the effective model featured on Fig. 4c,d at $k_{x}=\pi$; they are symmetry-enforced, hence their robustness to Rashba SOC. The two bands enclosed in the red rectangle, shown to the right of the V-point in Fig. 5a and to the left of the X-point in Fig. 5b, are twofold spin-degenerate. Therefore, when these two spin-degenerate bands stick together along the X-V edge of the BZ, they form a fourfold degenerate symmetry-enforced NL. The spin degeneracy is overall lifted in the presence of Rashba SOC, so each spin-degenerate band splits into two spin-split bands, as shown in Fig. 5c,d. In this case, the four non-degenerate bands to the right of the V-point in Fig. 5c and to the left of the X-point in Fig. 5d stick together pairwise to form two spin-polarized twofold degenerate symmetry-enforced NLs along the X-V edge. The NLs of Fig. 5g,h are our borophenes’ analogues of the NLs shown in Fig. 4d inside the BZ; despite being accidental, they survive in the regime of very strong Rashba SOC, so they must originate from the vacancy-induced anisotropy. Indeed, as shown in Fig. 5g,h, the effect of Rashba SOC of strength as large as $\lambda =0.1$ eV (way beyond the experimental bound) on the accidental NLs is just to lift their spin-degeneracy by shifting the spin-polarized bands in opposite directions in the $k_{x}-k_{y}$ plane. The accidental NLs inside the BZ are protected (i.e., enabled, but not enforced) by time-reversal symmetry, as manifest in their symmetric shape with respect to a momentum flip ${\textbf {k}}\rightarrow -{\textbf {k}}$ for the spin-degenerate bands in the absence of Rashba SOC, and with respect to a momentum-spin flip $({\textbf {k}},\uparrow )\rightarrow (-{\textbf {k}},\downarrow )$ for the spin-split bands in the presence of Rashba SOC. DFT calculations employing intrisic SOC (c.f. Sect. 4 of the Supporting Information) yield the same conclusion obtained for Rashba SOC, namely, robusteness of the edge and internal NLs to SOC. Our DFT results for vacancy-engineered borophenes thus confirm the existence of the two types of NLs predicted analytically by a minimal effective model containing only nearest-neighbor hopping. We note that the effective model entails a proof of principle, i.e., it demonstrates the existence of NLs based solely on the constraints that symmetries impose on a generic Hamiltonian. Complementarily, the first-principles calculations show that symmetry-preserving interactions, which are present in a real material, and material-specific details, such as the particular crystalline structure and chemical composition, do not hinder the NLs..

We note that the phenomena uncovered here are not exclusive to B$_{10}$ and B$_{16}$. We have found other borophenes with the same symmetries as B$_{10}$ and B$_{16}$^18^. These materials thus possess symmetry-enforced NLs and unusually robust accidental NLs in the spectrum. It is worth mentioning that the proposed structures fall within the stability range reported in Ref.^22^, $x\in [0.10,0.15]$, where *x* is a measure of concentration of vacancies.

## Discussion

We have shown how vacancy-engineering turns a monoatomic symmorphic 2D material into a nonsymmorphic one with a glide-plane symmetry. By carrying out a symmetry analysis and applying it to an effective model, we have demonstrated that the synthesized glide plane enforces two pairs of nodal points in the spectrum of the material. When the glide-plane symmetry is combined with inversion-point and time-reversal symmetries, the nodal points of each pair are connected through enforced NLs pinned at one edge of the BZ. We have also uncovered anisotropy-induced accidental NLs which can be moved around in the interior of the BZ by varying the anisotropic parameters, an interesting and potentially very useful byproduct of introducing vacancies.

DFT results for vacancy-engineered borophenes confirm the analytical predictions for the enforced NLs, and also convey that these materials have accidental NLs inside the BZ which survive under very strong Rashba SOC and intrinsic SOC. This enhanced robustness is consistent with an anisotropy-origin, and should be contrasted to the usual frailty of common accidental NLs. Besides the availability of various already realized borophenes with periodic distributions of vacancies^19–32^, another reason for choosing borophene as our test-material is the possible implications for topological superconductivity. Indeed, for an efficient braiding or fusion of Majorana states, necessary to utilize their non-Abelian statistics in topological quantum computing^37,38^, it is important to have a 2D topological superconductor. Such a 2D realization overcomes the need for fine-tuned material parameters to realize topological superconductivity in commonly studied 1D systems^39–41^, besides removing the 1D geometrical constraint, by that enabling braiding and fusion^42,43^. Since boron becomes superconducting when squeezed, fascinating prospects are thus opened starting from a borophene-made NLSM and enhancing its superconductivity by proximity effects and/or pressure. Finally, borophene is flexible and transparent, which might also encourage technological applications of our proposal.

For possible applications, enhanced robustness of the accidental NLs is particularly desirable. An accidental NL which is robust across the experimentally available ranges of perturbations’ strengths is at least as good as a symmetry-enforced one, with the advantage that the former can be moved around in the BZ. In fact, the enhanced robustness would allow the resulting NLSM to survive strong perturbations while being modified by various proximity effects^11^, at the same time that the ability to move the NLs might, conceivably, affect momentum-dependent scattering processes, or even suppresses them, which can be used to manipulate various susceptibilities of the material. Some guidance in such a design of nodal regions can be inferred from the example of unconventional superconductors: The change in the BZ location of the gap closing modifies the electric, magnetic, thermal, and optical responses of the material^44–48^. For example, in addition to the common Meissner effect, which is linear in the applied magnetic field, the presence of NLs is responsible for the nonlinear Meissner effect, whose angular dependence can then be modified by changing the position of the NLs. The presence of NLs and their location can be probed through the higher-harmonic generation for an applied harmonic magnetic field^49,50^. Finally, our proposal relies only on crystal symmetries, being applicable to general 2D materials, and possibly also to 3D materials^51^. The basic principle of creating a nonsymmorphic symmetry by introducing vacancies in a symmorphic crystal offers fresh opportunities for fundamental and applied research on NLSMs.

## Methods

The band structures of B$_{10}$ and B$_{16}$ are obtained by first-principles calculations using the QUANTUM ESPRESSO package^52^. The kinetic energies cutoff for wave function (ecutwfc) and for charge density (ecutrho) are set to 600 and 60 Ry, respectively. Perdew, Burke and Ernzerhof (PBE) form of the generalized gradient approximation (GGA) is adopted for the exchange-correlation energy^53^. Numerical integrations in the Brillouin zone are evaluated with the Monkhorst-Pack mesh of $10 \times 10 \times 1$. All structures are relaxed until the total energy converges to within $10^{-4}$ eV during the self-consistent loop, employing the Methfessele-Paxton method with a smearing of 0.2 eV width.

To incorporate Rashba SOC, we develop a tight-binding (TB) model using the package Wannier90^54^. This maps the ground-state wave functions from the density functional theory output file onto a maximally localized Wannier function basis $\{\vert {W}_{i\vec {R}}\rangle \}$, where $i=(I, \alpha )$ is the composite index accounting for the atom site $\vec {r}_I$ and the atomic orbital $\alpha$. $\vec {R}=R_{a}\vec {a}+R_{b}\vec {b}$ is the Bravais lattice vector with $R_{a/b}$ being the Bravais lattice vector component on the direction of the unit cell lattice vector $\vec {a}/\vec {b}$. The wave function is thus given by

5 $$\begin{aligned} \vert \phi _{i\vec {k}}\rangle = \frac{1}{\sqrt{N}}\sum _{\vec {R}}\,e^{i\vec {k}\cdot \vec {R}}\vert {W}_{i\vec {R}}\rangle . \end{aligned}$$

We employ an adaptive *k*-mesh strategy to build the TB Hamiltonian, $H_{TB}$, of borophene B$_{n}$ without Rashba SOC. The entries of $H_{TB}$ are given by

6 $$\begin{aligned}{}[H_{TB}(\vec {k})]_{ij} = \langle \phi _{i\vec {k}}\vert {H}_{TB}\vert \phi _{j\vec {k}}\rangle = \sum _{\vec {R}}e^{i\vec {k}\cdot \vec {R}}\,t_{ij}(\vec {R}), \end{aligned}$$

where $t_{ij}(\vec {R})$ = $\langle {W_{i0}}\vert {H_{KS}}\vert {W}_{j\vec {R}}\rangle$ is the matrix element extracted from the Wannier90 output file, with $H_{KS}$ being the Hamiltonian of Kohn–Sham equation.

Borophene B$_{n}$ subject to Rashba SOC is described by the Hamiltonian $H=H_{TB}+H_{R}$, where the Rashba SOC Hamiltonian:

7 $$\begin{aligned} H_{R} = \lambda (\sigma \times {p})\cdot \hat{z} = i\lambda \sum _{\langle {ij}\rangle ,s,s'}c_{is}^{\dag }(\sigma _{ss'}\times \mathbf{d}_{ij})^zc_{js'}, \end{aligned}$$

with *p* the momentum, $c_{is}^\dag$ ($c_{is}$) the creation (annihilation) operator of an electron of spin *s* at site *i*, $\mathbf{d}_{i,j}=\mathbf{r}_i-{\mathbf{r}_j}$ the vector connecting the pair of neighbor sites *i* and *j* in the lattice, $\sigma$ the vector of the Pauli matrices, and $\lambda$ the strength of the Rashba SOC, $\hat{z}$ the unit vector along z axis ^55^.

## Supplementary Information

Supplementary Information.

## Data Availability

The data generated and analysed during the current study is available from the corresponding author on reasonable request.
