# Supplementary material for: Vacancy-engineered nodal-line semimetals
Source: Sci Rep. 2022 Sep 2;12:14981. doi: 10.1038/s41598-022-18519-8 (PMC9630295; doi:10.1038/s41598-022-18519-8)
Supplement: Supplementary file 1 — Supplementary Information. [file 41598_2022_18519_MOESM1_ESM.pdf]

Supporting information
Vacancy-engineered nodal-line semimetals

Fujun Liu[1,2], Fanyao Qu[2], Igor Žutić[3], and Mariana Malard[4,*]

[1]Nanophotonics and Biophotonics Key Laboratory of Jilin Province, School of Physics, Changchun University of Science and Technology, Changchun, 130022, P.R.China
[2]Instituto de Física, Universidade de Brasília, Brasília-DF, 70904-910, Brazil
[3]University at Buffalo, the State University of New York, Buffalo, NY, 14260, USA
[4]Faculdade UnB Planaltina, Universidade de Brasília, Brasília-DF, 70904-910, Brazil
[*]Corresponding author. mmalard@unb.br

ABSTRACT

This Supporting Information contains the symmetry analysis of the glide-plane, inversion-point, and refection-plane symmetries of a 4×4 effective model describing a nonsymmorphic two-dimensional crystal, as well as density functional theory calculations for vacancy-engineered borophene sheets.

1 Glide-plane symmetry

Let $|k_x,k_y\rangle_\alpha$ ($\alpha=1,2,3,4$) be the eigenstate of the Bloch Hamiltonian $\mathscr{H}(k_x,k_y)$ with eigenenergy $E_\alpha(k_x,k_y)$, i.e.

$$\mathscr{H}(k_x,k_y)|k_x,k_y\rangle_\alpha = E_\alpha(k_x,k_y)|k_x,k_y\rangle_\alpha. \tag{S1}$$

Combining Eq. (1) and Eq. (S1) we arrive at

$$\mathscr{H}(k_x,k_y)\,\mathscr{G}(k_x,k_y)|k_x,-k_y\rangle_{\alpha'} = E_{\alpha'}(k_x,-k_y)\,\mathscr{G}(k_x,k_y)|k_x,-k_y\rangle_{\alpha'}, \tag{S2}$$

which means that $\mathscr{G}(k_x,k_y)|k_x,-k_y\rangle_{\alpha'}$ is an eigenstate of $\mathscr{H}(k_x,k_y)$ with eigenenergy $E_{\alpha'}(k_x,-k_y)$. It follows that $\mathscr{G}(k_x,k_y)|k_x,-k_y\rangle_{\alpha'}$ equals an eigenstate $|k_x,k_y\rangle_\alpha$ up to a phase, i.e.,

$$\mathscr{G}(k_x,k_y)|k_x,-k_y\rangle_{\alpha'} = e^{i\theta_{\alpha',\alpha}(k_x,k_y)}|k_x,k_y\rangle_\alpha. \tag{S3}$$

It also follows that $E_{\alpha'}(k_x,-k_y)=E_\alpha(k_x,k_y)$.

On the lines $k_y=\bar{k}_y=0,\pm\pi$, $\mathscr{H}(k_x,-\bar{k}_y)=\mathscr{H}(k_x,\bar{k}_y)$ (where $\mathscr{H}(k_x,\mp\pi)=\mathscr{H}(k_x,\pm\pi)$ follows from the 2π-periodicity of the BZ). On these lines, Eq. (1) yields $[\mathscr{G}(k_x,\bar{k}_y),\mathscr{H}(k_x,\bar{k}_y)]=0$, and hence $\mathscr{G}(k_x,\bar{k}_y)$ and $\mathscr{H}(k_x,\bar{k}_y)$ share a set of eigenstates. Indeed, substituting k_y by $\bar{k}_y$ in Eq. (S2) (and using that $\mathscr{H}(k_x,\bar{k}_y)=\mathscr{H}(k_x,-\bar{k}_y)$), we conclude that $\mathscr{G}(k_x,\bar{k}_y)|k_x,\bar{k}_y\rangle_{\alpha'}$ is an eigenstate of $\mathscr{H}(k_x,\bar{k}_y)$ with eigenenergy $E_{\alpha'}(k_x,\bar{k}_y)$. Since the eigenenergies are, in general, non-degenerate (i.e., $E_\alpha(k_x,k_y)\neq E_{\alpha'}(k_x,k_y)$ for $\alpha\neq\alpha'$), it follows that $\mathscr{G}(k_x,\bar{k}_y)|k_x,\bar{k}_y\rangle_{\alpha'}$ equals $|k_x,\bar{k}_y\rangle_{\alpha'}$ up to a phase, i.e.,

$$\mathscr{G}(k_x,\bar{k}_y)|k_x,\bar{k}_y\rangle_{\alpha'} = e^{i\theta_{\alpha'}(k_x)}|k_x,\bar{k}_y\rangle_{\alpha'}. \tag{S4}$$

The matrix $\mathscr{G}(k_x,k_y)$ can be obtained by extracting how the glide plane G transforms the second-quantized operator $c^j_{m_x,m_y}$ acting on the j-th site of the unit cell located at (m_x,m_y). Using Figure 2(a), it is easy to see that G acts on the operator of the unit cell at $(-1,-1)$ as

$$Gc^1_{-1,-1}=c^2_{-1,0} \qquad Gc^2_{-1,-1}=c^1_{0,0}$$
$$Gc^3_{-1,-1}=c^4_{-1,1} \qquad Gc^4_{-1,-1}=c^3_{0,1}$$

For an unit cell at $(-m_x,-m_y)$, G act as

$$Gc^1_{-m_x,-m_y}=c^2_{-m_x,m_y-1} \qquad Gc^2_{-m_x,-m_y}=c^1_{-m_x+1,m_y-1}$$
$$Gc^3_{-m_x,-m_y}=c^4_{-m_x,m_y} \qquad Gc^4_{-m_x,-m_y}=c^3_{-m_x+1,m_y}.$$

Applying the Fourier transform of $c^j_{m_x,m_y}$, i.e.

$$c^j_{m_x,m_y} = \sum_{k_x,k_y} c^j_{k_x,k_y} e^{i(k_x m_x + k_y m_y)},$$

we obtain these relations in momentum space as

$$G c^1_{k_x,k_y} = e^{ik_y} c^2_{k_x,-k_y} \qquad G c^2_{k_x,k_y} = e^{i(k_x+k_y)} c^1_{k_x,-k_y}$$
$$G c^3_{k_x,k_y} = c^4_{k_x,-k_y} \qquad G c^4_{k_x,k_y} = e^{ik_x} c^3_{k_x,-k_y}.$$

The above transformations can be carried out by applying the operator $\mathscr{G}(k_x,k_y) \Updownarrow_{k_y}$ to the spinor $c_{k_x,k_y} = [c^1_{k_x,k_y}\ \ c^2_{k_x,k_y}\ \ c^3_{k_x,k_y}\ \ c^4_{k_x,k_y}]^T$, where $\Updownarrow_{k_y}$ flips k_y and $\mathscr{G}(k_x,k_y)$ is the 4×4 matrix

$$\mathscr{G}(k_x,k_y) = \begin{bmatrix} e^{ik_y} g(k_x) & 0 \\ 0 & g(k_x) \end{bmatrix}, \qquad g(k_x) = \begin{bmatrix} 0 & 1 \\ e^{ik_x} & 0 \end{bmatrix}. \tag{S5}$$

The eigenvalues of $\mathscr{G}(k_x,\bar{k}_y)$ are found by solving the characteristic equation

$$\begin{aligned} \det[\mathscr{G}(k_x,\bar{k}_y) - \xi\,\mathbb{1}] &= \det \begin{bmatrix} \pm g(k_x) - \xi\,\mathbb{1} & 0 \\ 0 & g(k_x) - \xi\,\mathbb{1} \end{bmatrix} \\ &= \det[\pm g(k_x) - \xi\,\mathbb{1}] \det[g(k_x) - \xi\,\mathbb{1}] \\ &= (\xi^2 - e^{ik_x})^2 = 0. \end{aligned} \tag{S6}$$

The two-fold degenerate eigenvalues of $\mathscr{G}(k_x,\bar{k}_y)$ are thus

$$\xi_{1,3} = e^{ik_x/2},\ \xi_{2,4} = -e^{ik_x/2}. \tag{S7}$$

By enforcing Eq. (1), with $\mathscr{G}(k_x,k_y)$ given by Eq.(S5), the entries $\varepsilon_{n,m}(k_x,k_y)$ of $\mathscr{H}(k_x,k_y)$ get constrained by the relations:

$$\varepsilon_{n+1,n+1} = \varepsilon_{n,n},\ n = 1,3; \tag{S8}$$

$$\varepsilon^*_{n,n+1}(k_x,k_y) = e^{ik_x}\,\varepsilon_{n,n+1}(k_x,-k_y),\ n = 1,3; \tag{S9}$$

$$\varepsilon_{2,3}(k_x,k_y) = e^{i(k_x+k_y)}\,\varepsilon_{1,4}(k_x,-k_y); \tag{S10}$$

$$\varepsilon_{2,4}(k_x,k_y) = e^{ik_y}\,\varepsilon_{1,3}(k_x,-k_y). \tag{S11}$$

For the tight-binding model whose entries are given by Eq. (2) (with the specified conditions for the parameters given below Eq. (2)), Eqs. (S8)-(S11) yield

$$\varepsilon_{n+1,n+1} = \varepsilon_{n,n} = 2\mu_n,\ n = 1,3; \tag{S12}$$

$$\varepsilon_{n,n+1}(k_x,k_y) = 2i\,\mathrm{Im}(t_{n+1,n}) + t^*_{n+1,n}(1+e^{-ik_x}),\ n = 1,3; \tag{S13}$$

$$\varepsilon_{1,3}(k_x,k_y) = t^*_{3,1} + t_{1,3}(1+e^{ik_y}); \tag{S14}$$

$$\varepsilon_{1,4}(k_x,k_y) = t_{1,4} + t^*_{4,1}(1+e^{-ik_x}); \tag{S15}$$

$$\varepsilon_{2,3}(k_x,k_y) = e^{i(k_x+k_y)}\,[t_{1,4} + t^*_{4,1}(1+e^{-ik_x})]; \tag{S16}$$

$$\varepsilon_{2,4}(k_x,k_y) = e^{ik_y}\,[t^*_{3,1} + t_{1,3}(1+e^{-ik_y})]; \tag{S17}$$

and $\varepsilon_{m,n}(k_x,k_y) = \varepsilon^*_{n,m}(k_x,k_y)$.

2 Inversion-point symmetry

A similar procedure to the one outlined in Section 1 for G yields the operator $\mathscr{I}(k_y)\Updownarrow_{k_x,k_y}$ describing the inversion-point transformation I, with

$$\mathscr{I}(k_y) = \begin{bmatrix} e^{ik_y}\sigma_x & 0 \\ 0 & \sigma_x \end{bmatrix}, \qquad \sigma_x = \begin{bmatrix} 0 & 1 \\ 1 & 0 \end{bmatrix}. \tag{S18}$$

The constraints imposed by Eq. (3), with $\mathscr{I}(k_y)$ given by Eq.(S18), on the entries $\varepsilon_{n,m}(k_x,k_y)$ of $\mathscr{H}(k_x,k_y)$ are

$$\varepsilon^*_{n,n+1}(k_x,k_y) = \varepsilon_{n,n+1}(-k_x,-k_y), n = 1,3; \tag{S19}$$

$$\varepsilon_{2,3}(k_x,k_y) = e^{ik_y}\,\varepsilon_{1,4}(-k_x,-k_y); \tag{S20}$$

$$\varepsilon_{2,4}(k_x,k_y) = e^{ik_y}\,\varepsilon_{1,3}(-k_x,-k_y). \tag{S21}$$

Applying Eqs. (S19)-(S21) to the tight-binding model with entries given by Eq. (2) (with the specified conditions for the parameters given below Eq. (2)) leads to

$$\varepsilon_{n,n+1}(k_x,k_y) = t_{n,n+1} + t_{n+1,n}(1+e^{-ik_x}), n = 1,3; \tag{S22}$$

$$\varepsilon_{1,3}(k_x,k_y) = t^*_{3,1} + t_{1,3}(1+e^{ik_y}); \tag{S23}$$

$$\varepsilon_{1,4}(k_x,k_y) = t_{1,4} + t^*_{4,1}(1+e^{-ik_x}); \tag{S24}$$

$$\varepsilon_{2,3}(k_x,k_y) = e^{ik_y}\,[t_{1,4} + t^*_{4,1}(1+e^{ik_x})]; \tag{S25}$$

$$\varepsilon_{2,4}(k_x,k_y) = e^{ik_y}\,[t^*_{3,1} + t_{1,3}(1+e^{-ik_y})], \tag{S26}$$

with $t_{n,n+1}$ and $t_{n+1,n} \in \Re$, $n = 1,3$, and $\varepsilon_{m,n}(k_x,k_y) = \varepsilon^*_{n,m}(k_x,k_y)$.

Pairwise combining Eqs. (S13)-(S17) and Eqs. (S22)-(S26) yields the off-diagonal entries of the glide-plane and inversion-point invariant tight-binding model:

$$\varepsilon_{n,n+1}(k_x,k_y) = t_{n+1,n}(1+e^{-ik_x}); \tag{S27}$$

$$\varepsilon_{1,3}(k_x,k_y) = t^*_{3,1} + t_{1,3}(1+e^{ik_y}); \tag{S28}$$

$$\varepsilon_{1,4}(k_x,k_y) = t^*_{4,1}(1+e^{-ik_x}); \tag{S29}$$

$$\varepsilon_{2,3}(k_x,k_y) = e^{ik_y}\,[t^*_{4,1}(1+e^{ik_x})]; \tag{S30}$$

$$\varepsilon_{2,4}(k_x,k_y) = e^{ik_y}\,[t^*_{3,1} + t_{1,3}(1+e^{-ik_y})], \tag{S31}$$

with $t_{n+1,n} \in \Re$, $n = 1,3$. The diagonal entries are given by Eq. (S12).

3 Reflection-plane symmetry

A similar procedure to the one outlined in Section 1 for G yields the operator $\mathscr{R}(k_x) \Updownarrow_{k_x}$ describing the reflection-plane transformation R, with

$$\mathscr{R}(k_x) = \mathscr{G}(k_x,k_y)\mathscr{I}(-k_y), \tag{S32}$$

and $\mathscr{G}(k_x,k_y)$ and $\mathscr{I}(k_y)$ given by Eqs. (S5) and (S18), respectively.

Rewriting Eq. (3) as $\mathscr{I}(-k_y)\mathscr{H}(-k_x,k_y)\mathscr{I}^{-1}(-k_y)$=$\mathscr{H}(k_x,-k_y)$ and inserting that into Eq. (1), we get:

$$\mathscr{G}(k_x,k_y)\mathscr{I}(-k_y)\mathscr{H}(-k_x,k_y)\mathscr{I}^{-1}(-k_y)\mathscr{G}^{-1}(k_x,k_y) = \mathscr{H}(k_x,k_y) \tag{S33}$$

Now, using Eq. (S32), the latter expression yields:

$$\mathscr{R}(k_x)\mathscr{H}(-k_x,k_y)\mathscr{R}^{-1}(k_x) = \mathscr{H}(k_x,k_y), \tag{S34}$$

which is the invariance relation of $\mathscr{H}(k_x,k_y)$ with respect to $\mathscr{R}(k_x)$. That is, if the glide plane G and the inversion point I are symmetries, then the reflection plane R is also a symmetry.

4 DFT with intrinsic SOC

Intrinsic SOC is described by the Hamiltonian

$$H_{ISOC} = \lambda_I(\vec{L}\cdot\vec{S}), \tag{S35}$$

where $\vec{L}$ and $\vec{S}$ are the orbital angular momentum and spin angular momentum operators, respectively, and λ_I is the strength of the intrinsic SOC. To study the effect of intrinsic SOC on the NLs in the band structure of a vacancy-engineered borophene we use full-relativistic pseudopotentials in the DFT calculation. Like Rashba SOC, intrinsic SOC partially lifts the degeneracy of the bands. However, since boron is a very light atom, the intrinsic SOC-induced band-splitting is only few meVs in energy and, unlike Rashba SOC-induced band-splitting, cannot be magnified through external control of an electric field. Figure S1(a) shows the band structure of B_{10} in the presence of intrinsic SOC, with the amplified image of the bands inside the red rectangle shown in Figure S1(b). The latter demonstrates the existence of two split NLs along the X-V edge of the BZ. Each one of these two-fold degenerate NLs separates into non-degenerate bands at the V-point, i.e., upon exiting the BZ edge. In contrast, without intrinsic SOC, the NLs along the BZ edge are four-fold degenerate, and separate into two-fold degenerate bands when exiting the BZ edge. In conclusion, while half of the degeneracy can be lifted by intrinsic SOC, the remaining two-fold degeneracy which is symmetry-enforced for the edge NLs cannot.

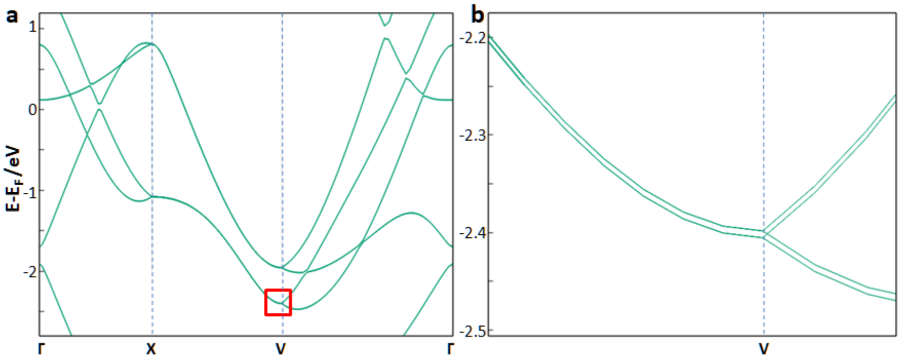

Figure S1. (a) Band structure of B_{10} with intrinsic spin-orbit coupling along the Γ-X-V-Γ path in the Brillouin zone. (b) Zoomed-in band structure inside the red rectangle indicated in panel (a).
